# Multiple Sclerosis–Specific Reference Curves for Brain Volumes to Explain Disease Severity

**DOI:** 10.1212/WNL.0000000000213618

**Published:** 2025-04-23

**Authors:** David Rudolf van Nederpelt, Lonneke Bos, Rozemarijn M. Mattiesing, Eva M.M. Strijbis, Bastiaan Moraal, Joost Kuijer, Jeroen Hoogland, Henk J.M.M. Mutsaerts, Bernard Uitdehaag, Joep Killestein, Lizette Heine, Bas Jasperse, Frederik Barkhof, Menno M. Schoonheim, Hugo Vrenken

**Affiliations:** 1 MS Center Amsterdam, Radiology and Nuclear Medicine, Vrije Universiteit Amsterdam, Amsterdam Neuroscience, Amsterdam UMC location VUmc, the Netherlands;; 2 MS Center Amsterdam, Neurology, Vrije Universiteit Amsterdam, Amsterdam Neuroscience, Amsterdam UMC location VUmc, the Netherlands;; 3 Department of Epidemiology and Data Science, Amsterdam UMC, the Netherlands;; 4 Quantib B.V., DeepHealth, Rotterdam, the Netherlands;; 5 UCL London, Institutes of Neurology and Healthcare Engineering, London, United Kingdom; and; 6 MS Center Amsterdam, Anatomy and Neurosciences, Vrije Universiteit Amsterdam, Amsterdam Neuroscience, Amsterdam UMC location VUmc, the Netherlands.

## Abstract

**Background and Objectives:**

Brain atrophy is relevant for understanding disease progression and treatment response in people with multiple sclerosis (pwMS). Automatic brain volume–reporting tools often rely on healthy control (HC) reference curves to interpret brain volumes, whereas brain volume loss is different in pwMS. This observational study aimed to develop an MS-specific reference model for brain volumes and evaluate its performance compared with HC-based curves, as a proof-of-concept.

**Methods:**

Participants, pwMS and HCs, from the Amsterdam MS cohort were included based on the availability of T1-weighted MR scans. Normalized brain volumes (NBVs) were obtained using commercially available software. The software program also provides NBV percentiles, based on age-specific and sex-specific HC curves, grouped into NBV quartiles, describing deviation from expected NBVs. Disease severity was determined with the MS severity score (MSSS), Symbol Digit Modalities Test (SDMT), and 9-Hole Peg Test (9HPT). An MS-specific model was developed by regressing NBVs against age, sex, disease duration, and MS phenotype. The resulting MS model was also used to classify pwMS into quartiles describing deviation from expected NBV, given the modeled patient characteristics, with leave-one-out predictions. Quartile classification from HC-based and MS-based reference curves was compared with MSSS using analysis of variance (ANOVA).

**Results:**

Regressions for NBVs from 713 pwMS and 259 HCs (mean age: 49.1 ± 9.7 and 48.3 ± 10.1, %female: 70.4% and 67.2%, respectively) were significant for age, sex, disease duration, and phenotype, which were included in the MS-specific model. MS-specific model quartile designations significantly improved associations with MSSS values (*p* = 2.2*10^−9^, *η*^2^ = 0.06) compared with HC-based quartiles. MSSS values worsened with lower NBV quartiles in the MS-specific model (difference between quartiles 1–4 = −0.84, *p* = 6.1*10^−3^, 95% CI [−1.5 to −0.18])), which was not observed for HC-based quartiles (*p* = 0.98). Quartile group differences were observed for 9HPT (MS: *p* = 3.5*10^−3^, *η*^2^ = 0.02, HC: *p* = 6.6*10^−3^, *η*^2^ = 0.02) and SDMT (MS: *p* = 3.1*10^−4^, *η*^2^ = 0.05, HC: *p* = 5.4*10^−4^, *η*^2^ = 0.04) values, but MS-specific quartiles again improved quartile associations (*p* = 0.036, *η*^2^ = 0.01 and *p* = 0.02, *η*^2^ = 0.01, respectively).

**Discussion:**

NBV values derived from an MS-specific reference model offer improved relevance for assessing disease severity compared with curves derived from age-specific and sex-specific HC reference models. Improving the model toward application in individual people could enhance clinical implementation.

## Introduction

Multiple sclerosis (MS) is a neurologic condition affecting the CNS, leading to both focal inflammatory lesions and widespread neurodegeneration.^1^ While the impact of this disease on patients is well recognized, current clinical routine often lacks comprehensive tools to accurately predict disease progression and treatment efficacy.^2^ Clinical evaluation of people with MS (pwMS) currently focuses on lesion detection and quantification, which is relevant for diagnosis and concurrent disease activity. The clinical potential of brain atrophy measurements is increasingly recognized as well because it mirrors the neurodegenerative aspect of MS.^3^ Atrophy in pwMS can start from the onset of the disease and is worse compared with normal aging.^4,5^ Furthermore, it has been shown to predict clinical progression to a greater extent than lesion measures.^6^ Currently available advanced treatments are highly successful at halting the inflammatory component of MS; however, clinical progression in pwMS remains common and relates to neurodegeneration, which makes slowing the neurodegenerative component currently the most important novel target.^7^

Despite advancements in MS treatments that show potential in attenuating both lesion accrual and brain atrophy rates,^8^ monitoring treatment efficacy based on atrophy rates remains difficult. Radiologic evaluation of the impact of MS predominantly relies on visual assessment, lacking the depth required for precise and quantitative measurements.^3^ This gap in clinical translation of MRI capabilities poses a considerable challenge in optimizing treatment strategies for individual pwMS. In recent years, the development of commercial quantitative radiologic reporting tools for MS has rapidly increased.^9^ These tools offer automated analysis of MRI scans, quantifying brain and lesion volumes, often contextualized with reference data. This approach could provide clinicians and patients with valuable insights into the severity of disease. However, the reference data used in such tools are often based on a healthy control (HC) population, focusing on sex and age only. However, atrophy in patients with MS is more severe and occurs earlier than in a healthy control population, which could make traditional references inadequate for detecting the nuanced changes related to the disease progression and treatment impact. We, therefore, hypothesize that such a reference is insufficient because it does not account for MS-specific individual variation and treatment effects.

To increase the usability of brain atrophy measurement interpretation, this study aims to investigate whether disease severity can be explained better using MS-specific curves of neurodegeneration compared with general HC-based curves and how such MS-specific curves should be established. The study aims to establish a proof-of-concept and, if successful, these curves could then be refined for clinical implementation.

## Methods

### Participants

From the Amsterdam MS cohort, participants were retrospectively included based on the availability of a precontrast high-resolution (i.e., ≤1 mm^3^ isotropic resolution) 3D T1-weighted (T1w) MRI scan. Participants had to be 18 years or older and either were healthy controls (HCs) or had to have a diagnosis of relapsing remitting MS (RRMS), secondary progressive MS (SPMS), or primary progressive MS (PPMS).

### Standard Protocol Approvals, Registrations, and Patient Consents

The study protocol was approved (2020.269) by the Medical Research Ethics Committee of the Amsterdam UMC, and every participant gave written informed consent according to the Declaration of Helsinki.

### MRI

Cross-sectional MRI scans were performed as reported previously.^5,10-15^ In short, all participants were scanned on a 3T whole-body magnetic resonance system (GE Signa-HDxt/Discovery MR750, General Electric, Milwaukee, WI) using the same 8-channel phased-array head coil. Data were included from a combination of a subcohort of participants; some subcohorts were scanned on the Signa-HDxt and some on the Discovery MR750 with highly similar protocols. All the participants underwent 3D-T1w imaging, implemented as an inversion recovery fast spoiled gradient-echo (IR-FSPGR) sequence (repetition time [TR]: 8 ms, echo time [TE]: 3 ms, inversion time [TI]: 450 ms, flip angle (FA) 12°, 1.0 × 0.9 × 0.9 mm^3^ voxel size) for participants scanned on the Signa-HDxt and a similar IR-FSPGR (TR: 8.2 ms, TE: 3.2 ms, TI: 450 ms, FA: 12°, 1 × 1 × 1 mm^3^ voxel size) for participants scanned on the Discovery MR750.

### Clinical Measures

Clinical measures included basic demographic information (age and sex); disease duration (defined as time since symptom onset); history of disease-modifying therapy (DMT) use; and, when available, the efficacy of DMT categorized as low, medium, or high. Furthermore, measures of the Expanded Disability Status Scale (EDSS), the Timed 25-Foot Walk Test (T25FW), 9-Hole Peg Test (9HPT), and Symbol Digit Modalities Test (SDMT), when available and performed on the same day of MRI, were collected. To assess global disease severity, the MS severity score (MSSS) was calculated.^16^ The MSSS was chosen because it reflects deviation in disability compared with peers similar to the brain volume reference curves. Because disease duration influences both brain volume and disability, this was taken into account for both. Furthermore, we included clinical testing from participants who had a clinical-only 5-year follow-up, without MRI. For this subset, EDSS score worsening or improvement was defined as a 1.5-point increase if baseline EDSS score was 0.0, a 1.0-point change if baseline EDSS score was ≤4.5, and a 0.5-point change if EDSS score was >4.5.^17^ Next to that, cognition was assessed using an expanded brief repeatable battery of neuropsychological tests, with reliable change index (RCI) cutoffs derived from healthy control performance over 5 years, as described and defined previously.^18,19^ Cognitive decline was defined as meeting a 90% confidence RCI threshold on at least 2 tests. This approach minimizes false positives by applying stringent criteria across multiple neuropsychological tests.

### Volumetric Assessment on MRI

For volumetric assessment, commercially available software Quantib ND version 2.1 (Quantib BV, Rotterdam, The Netherlands) was used. Quantib ND calculates volumes of regions on 3D T1w scans. In addition, the intracranial volume (ICV) is computed by combining the regions of brain tissue and CSF. The segmentation algorithm has previously been described.^20,21^ The software program provides brain volumes as absolute values in cm^3^ and percentage of the ICV (brain relative volume). The latter is used for comparison against the reference population. Quantib ND does not require lesion filling.

### HC-Based Curves

In addition to volumetric assessment, the version of Quantib ND used also provides the brain volume percentile with reference to a large population of HCs. The reference population consists of an external cohort of almost 6,000 nondemented participants aged between 18 and 95 years, which included various public brain MRI data sets and HCs from the Rotterdam Scan Study.^22,23^ This included 2,782 women (51.2%) and 2,651 men (48.8%). Subsequently, pwMS were grouped into quartiles based on brain percentiles derived from Quantib ND product reference curves and from our MS-based model (as described in the next section). Brain percentiles reflect the relative position of an individual's brain volume compared with the reference population, allowing for categorization into quartiles for further analysis. Cutoff points for conversion of percentiles to quartiles were 25, 50, and 75 percent. Quartiles rather than percentiles were specifically chosen for this proof-of-concept study, given the limited sample size used to create the MS-specific model, because quartiles provide a practical balance between stratification detail and maintaining sufficient data within each group for reliable statistical analysis.^24^

### MS-Specific Model

As previously mentioned, quantifying MS-specific reference curves would require an MS-specific model. To contrast the performance of the HC-based model with an MS-specific model, we first investigated which factors to include in the MS-specific curves. Brain relative volumes provided by Quantib ND in both pwMS and HCs were regressed on age, sex, disease duration, and phenotype (HC/RRMS/SPMS/PPMS) using stepwise linear regression. These regression coefficients, therefore, provided estimates of the impact of these variables, which were then used to model MS-specific brain volume variation. Key assumptions for this model include normality of residuals, linearity of the covariate effects, and absence of interactions between the covariates.

After formulating this model, we, therefore, explored possible non-normal distributions, nonlinearity, and interaction effects. Possible improvements of the linear regression model were explored using beta-regression (i.e., to cope with possible non-normality) and nonlinear covariate effects modeled as smoothing splines. Furthermore, the additive value of all relevant two-way interactions was explored using a recently proposed hierarchical Bayesian shrinkage method (linked shrinkage) that adequately controls overfitting when exploring covariate interactions in this setting with the number of cases still considerably larger than the number of model parameters.^25^ The specified interactions were between age and sex for controls and all two-way interactions for participants with MS. Data fitting of all models was compared using the Akaike information criterion (AIC), prediction performance, and leave-one-out cross-validation where appropriate. Uncertainty regarding the estimated parameters in the final model was gauged through bootstrapping with 2,500 iterations, yielding 95% CIs.

### Statistical Analyses

Statistical analyses were conducted using R Statistical Software (version 4.0.3; R Foundation for Statistical Computing, Vienna, Austria).

The distribution of variables was evaluated using the Kolmogorov-Smirnov test and histogram inspection. For demographic variables, differences between groups (HC, RRMS, SPMS, PPMS) were examined using analysis of variance (ANOVA) for normally distributed variables with Tukey HSD post hoc tests for pairwise comparisons, Kruskal-Wallis tests for non-normally distributed data with Dunn tests for post hoc comparisons, and Chi-square tests for categorical variables. Heteroscedasticity was assessed using the Levene test for equal variances.

### Added Value of MS-Specific Reference Model

The outcome measure (quartile) was a grouping of individual estimates of how much the measured brain volume deviates from the predicted brain volume as an indication of the severity of atrophy at baseline, based on either the HC-based or the MS-specific model. To mitigate concerns on circularity for the MS-specific model estimates, individual patients were divided into quartiles using leave-one-out predictions.

Using the HC-based quartiles, between-quartile differences were related to MSSS using ANOVA and Tukey HSD post hoc tests when the main effect of the group (quartile) was significant. Subsequently, the procedure was repeated for the MS-specific quartiles to assess whether MS-specific curves showed similar differences between groups. Next, to specifically quantify the added value of MS-specific quartiles beyond the HC-based quartile definition, first the effect of HC-based quartiles on the MSSS was assessed using a linear regression model. Subsequently, the MS-specific quartile definition variable was added in the same model. The difference between the 2 models was tested using ANOVA. In addition, subgroup analyses were conducted separating patients with RRMS and progressive MS, based on that different immunopathologic mechanisms might drive brain atrophy in these subgroups. While the reference models already account for heterogeneity across MS subtypes, these analyses aimed to explore potential differences in clinical outcomes specifically in relation to MSSS. Because DMTs can influence brain volumes, we conducted χ^2^ tests between the quartiles for DMT use ever (yes or no) and efficacy (low, medium, and high).^26,27^

Furthermore, we conducted the same analyses to explore relationships with the T25FWT, 9HPT, and SDMT scores, correcting for age, disease duration, sex, and MS phenotype. In addition, we investigated whether the baseline quartile assignment could serve as a predictor for 5-year follow-up clinical variables, with baseline value and follow-up time as covariates. Moreover, differences between quartiles regarding EDSS worsening, stability, or improvement were assessed with multinominal logistic regression. This was also performed for cognitively stable or declining with logistic regression. Both were corrected for follow-up time.

*p* Values <0.05 (corrected, when applicable) were considered significant.

### Data Availability

Data may be shared (pseudonymized) at the request of any qualified investigator for purposes of replicating procedures and results.

## Results

### Demographics

**Figure 1:**
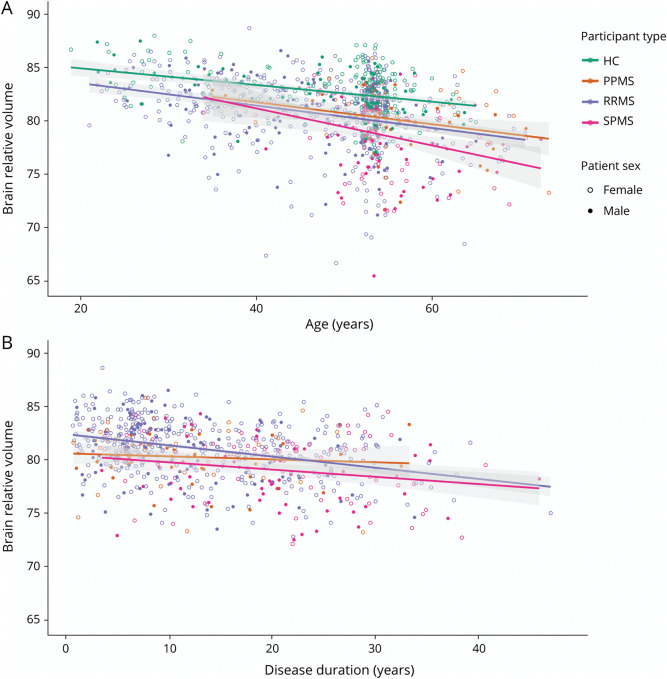
Scatterplot of Brain Relative Volume Plotted Against Age and Disease Duration Scatterplot of brain relative volume plotted against age. Regression lines are modeled for brain relative volume as function of age per participant type (HC, PPMS, RRMS, SPMS) (A). Scatterplot of brain relative volume plotted against disease duration. Regression lines are modeled for brain relative age as function of disease duration per participant type (B). HC = healthy control; PPMS = primary progressive multiple sclerosis; RRMS = relapsing remitting multiple sclerosis; SPMS = secondary progressive multiple sclerosis.

Baseline characteristics for the different phenotypes and HC groups are summarized in Table 1. The sample consisted of 713 pwMS with clinically definite MS and 259 HCs. Among the pwMS, 503 were diagnosed with RRMS, 134 with SPMS, and 76 with PPMS. A subset of 297 pwMS had a clinical-only 5-year follow-up (mean follow-up time: 4.93 ± 0.72 years) without MRI at follow-up. From this subset, 23 patients converted from RRMS to SPMS between the baseline and follow-up visit resulting in 230 to 207 participants with RRMS, 45 to 68 with SPMS, and 22 with PPMS. As expected, symptom duration was longer (*p* = 2.24 * 10^−19^) for SPMS compared with RRMS (difference = 7.9 years, 95% CI [5.9–9.9], *p* < 0.001) and SPMS compared with PPMS (difference = 8.4 years, 95% CI [5.5–11.3], *p* < 0.001). Moreover, EDSS scores were higher (*p* = 1.41 * 10^−41^), for SPMS compared with RRMS and PPMS compared with RRMS (*z* = −12.3, *p* = 1.3 * 10^−34^, *z* = 8.0, *p* = 1.6 * 10^−15^, respectively). Brain relative volume provided by Quantib ND was lower for all MS phenotypes compared with HCs and for participants with SPMS compared with RRMS and PPMS groups. Scatter plots for effects of age and disease duration on brain relative volume are demonstrated in Figure 1.

**Table 1:** Demographic, Clinical, and MRI Data

	HCs	RRMS	SPMS	PPMS	Overall *p* value
Participants, n	259	503	134	76	
Age (range), y	48.3 ± 10.1 (18.9–65.0)	46.7 ± 9.8 (21.0–70.6)^a^	54.5 ± 5.6 (34.0–72.4)^b^	55.8 ± 7.6 (35.0–73.3)^c,d,e^	<0.01^f^
F/M, n (%female)	174/85 (68)	389/114 (77)^a^	78/56 (56)^g^	35/41 (48)^c,d^	<0.01^h^
Symptom duration (range), y		12.8 ± 8.7 (0.69–47.0)	20.7 ± 8.9 (3.5–45.9)^g^	12.3 ± 8.2 (0.7–33.3)^d^	<0.01^i^
EDSS score (range)		2.9 ± 1.2 (0–7.5)	5.0 ± 1.7 (1.0–8.5)^g^	4.6 ± 1.6 (0–8.0)^d^	<0.01^f^
Brain relative volume %ICV (range)	82.7 ± 2.2 (76.5–88.0)	80.8 ± 3.3 (66.7–90.9)^a^	78.7 ± 3.5 (65.5–84.6)^b,g^	79.9 ± 3.3 (64.6–85.9)^c,e^	<0.01^i^
DMT ever (yes/no/NA)		261/116/126	66/29/39	15/47/15	<0.01^h^
DMT efficacy during scan (low/middle/high/none/NA)		111/10/27/229/126	18/3/6/68/39	2/0/7/53/14	<0.01^h^

Abbreviations: EDSS = Expanded Disability Status Scale; HCs = healthy controls; NA = not available; PPMS = primary progressive multiple sclerosis; RRMS = relapsing remitting multiple sclerosis; SPMS = secondary progressive multiple sclerosis.

a Significant difference between HCs and those with RRMS.

b Significant difference between HCs and those with SPMS.

c Significant difference between HCs and those with PPMS.

d Significant difference between RRMS and PPMS groups.

e Significant difference between SPMS and PPMS groups.

f Kruskal-Wallis test.

g Significant difference between RRMS and SPMS groups.

h Chi-square test.

i Analyses of variance.

### MS-Specific Brain Volume Reference Model

**Figure 2:**
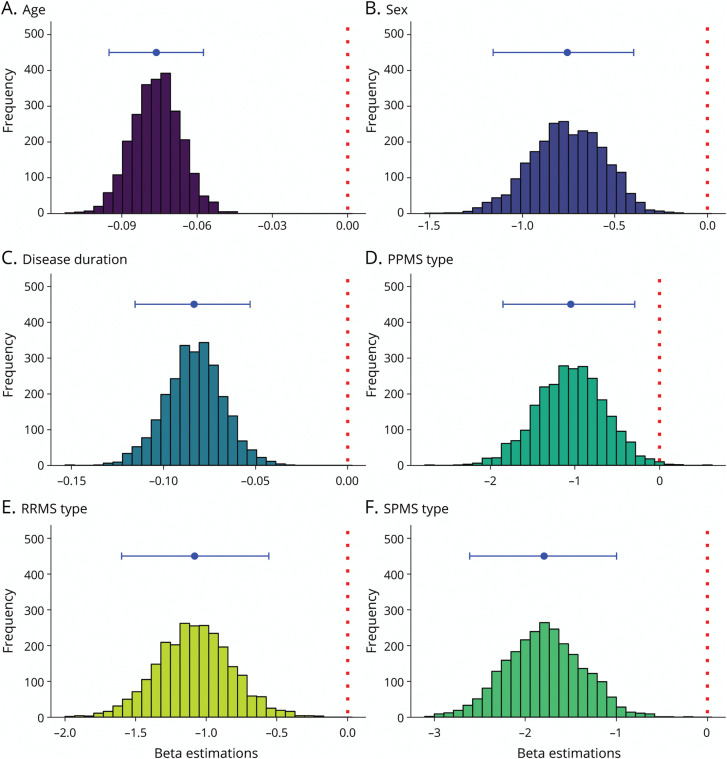
Histograms of Bootstrapped Beta Coefficients for Each Predictor Histograms of the beta coefficients gauged through bootstrapping, the red dotted line indicates zero, and the blue point and lines indicate the model's beta and the 95% CIs for regression of age (A), sex (male vs female) (B), disease duration (C), PPMS type vs HC (D), RRMS type vs HC (E), and SPMS type vs HC (F). Note that the x-axis is different for each histogram. HC = healthy control; PPMS = primary progressive multiple sclerosis; RRMS = relapsing remitting multiple sclerosis; SPMS = secondary progressive multiple sclerosis.

**Figure 3:**
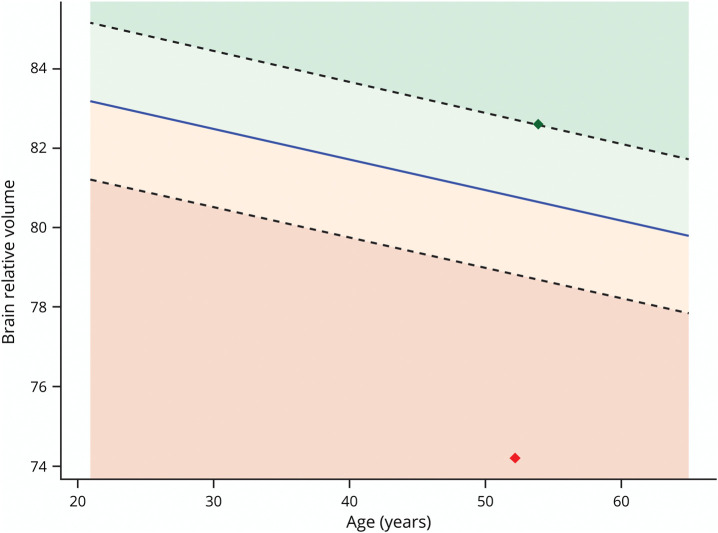
Example of MS-Based Reference Curves for Female Patients With RRMS With a Disease Duration of 10 Years Example of reference curves for female participants with RRMS with a disease duration of approximately 10 years. Red indicates the first quartile, orange the second quartile, light green the third, and green the fourth. The green dot represents a person who is 54.0 years old, has a disease duration of 10.5 years, and has an MSSS of 1.73. She is in the fourth quartile while the red dot in the first quartile represents a patient who is 52.2 years old and has a disease duration of 9.34 years with an MSSS of 7.18. MS = multiple sclerosis; MSSS = multiple sclerosis severity score; RRMS = relapsing remitting multiple sclerosis.

The stepwise linear regression analysis revealed significant associations (*R*^2^ = 0.265, adjusted *R*^2^ = 0.261, *F*(6, 965) = 58.1, *p* < 2.2 * 10^−16^) between brain relative volume and age (*β* = −0.077, 95% CI [−0.1 to −0.6], *p* = 3.1 * 10^−12^), patient sex (male vs female, *β* = −0.758, 95% CI [−1.2 to −0.4], *p* = 2.3* 10^−4^), disease duration (*β* = −0.083, 95% CI [−0.1 to −0.6], *p* = 1.1 * 10^−9^), and MS phenotype, including PPMS participants vs HCs (*β* = −1.038, 95% CI [−1.8 to −0.2], *p* = 0.01), RRMS sample vs HCs (*β* = −1.076, 95% CI [−1.6 to −0.5], *p* = 1.7 * 10^−4^), and SPMS sample vs HCs (i = −1.787, 95% CI [−2.6 to −1.0], *p* = 1.1 * 10^−5^). There were no meaningful nonlinear effects (eFigure 1). However, residuals of the model were somewhat skewed to the left (eFigure 2). Switching to beta-regression slightly improved AIC (−4,197 vs −4,132) and again revealed linear covariate contributions (eFigure 3). The slight differences between models mostly related to predictions for the lowest 10% of brain volumes, and hence, quartile group predictions were nearly identical (eFigure 4). Finally, linked shrinkage showed that all interactions failed to contribute substantively, with posterior estimates centered around zero. In summary, extensive checks on the assumption underlying the simple linear main effects did not reveal any need for modifications for our prediction purposes. For the final model, the 95% CIs of all coefficients as obtained by bootstrapping analyses excluded 0 and are demonstrated in Figure 2 together with the histograms. An example of the MS-based reference curves is illustrated in Figure 3. There were no differences in DMT use ever (*p* = 0.39) or efficacy (*p* = 0.77) between the quartiles for both MS-specific and HC-based quartiles (eFigures 5 and 6). A detailed overview of DMT use is provided in eTable 1.

### Cross-Sectional Relation to Baseline Clinical Outcome Measures

**Figure 4:**
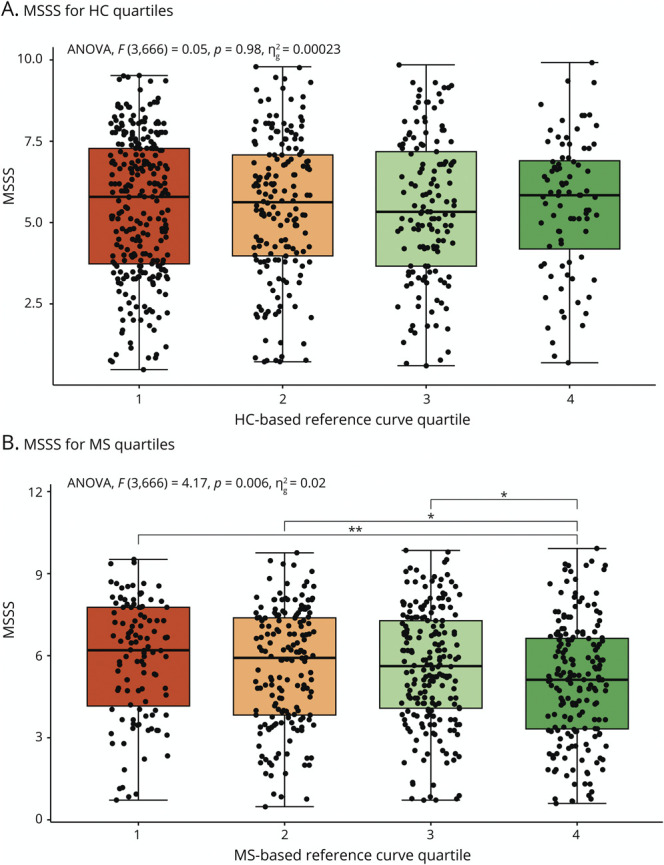
MSSS for Quartiles Derived From Both HC-Based and MS-Specific Models MSSS for the quartiles derived from the HC-based reference curves (A) and the MS-specific reference curves (B). *p* Values are derived from the Tukey HSD test. * = *p* < 0.1 and > 0.05, ** = *p* < 0.01. HC = healthy control; MS = multiple sclerosis; MSSS = multiple sclerosis severity score.

**Figure 5:**
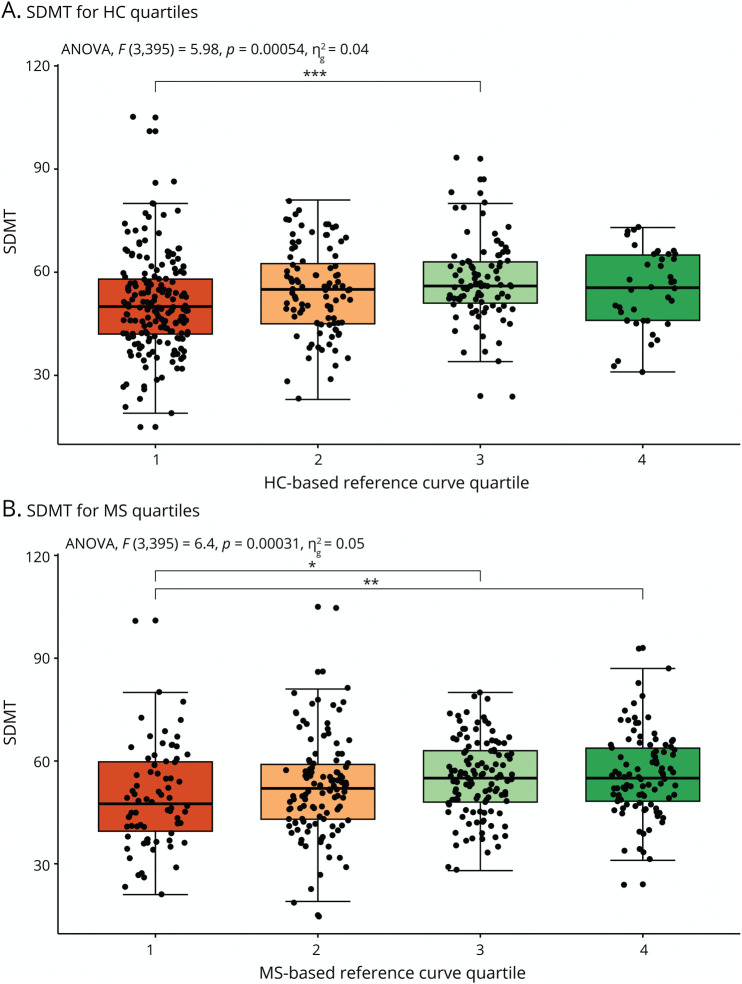
SDMT Scores for Quartiles Derived From Both HC-Based and MS-Specific Models SDMT scores for the quartiles derived from the HC-based reference curves (A) and the MS-specific reference curves (B). *p* Values are derived from the Tukey HSD test. * = *p* < 0.05, ** = *p* < 0.01, *** = *p* < 0.001. HC = healthy control; MS = multiple sclerosis; SDMT = Symbol Digit Modalities Test.

The overall test for MSSS differences between the quartile groups obtained from the MS-specific curves was significant (*p* = 6.0*10^−3^, *η*^2^ = 0.02) while the overall test was not significant for the HC-based reference curves (*p* = 0.98, *η*^2^ = 2.3*10^−4^). Post hoc testing revealed differences between the fourth and first quartile (difference = −0.84, *p* = 6.1*10^−3^, 95% CI [−1.5 to −0.18]), and a trend was observed between the fourth and second quartile (difference = −0.53, *p* = 0.09, 95% CI [−1.1 to 0.06]) and the fourth and third quartile (difference = −0.54, 95% CI [−1.1 to 0.01], *p* = 0.06) (Figure 4). MSSS was higher for the lower MS-specific quartiles, indicating more severe disease for pwMS who have decreased brain relative volumes compared with the MS-specific reference population. In addition, MS-specific reference model quartiles improved associations with MSSS values (*p* = 2.2 * 10^−9^, *η*^2^ = 0.059) compared with quartiles obtained from HC reference curves. EDSS distribution across quartiles is further demonstrated in eFigure 7. No differences were observed between RRMS and progressive MS groups regarding correlations with MSSS, apart from variations in post hoc analyses (eFigures 8 and 9). The 9HPT scores for both the MS-specific and HC-based quartiles were different between quartile groups (*p* = 3.5*10^−3^, *η*^2^ = 0.022, and *p* = 6.6*10^−3^, *η*^2^ = 0.020, respectively), but MS-specific quartiles improved quartile associations with the 9HPT (*p* = 0.036, *η*^2^ = 0.007). For the HC-based quartiles, 9HPT values of participants in quartile 1 were significantly higher than of participants in quartile 2 (difference = −2.1, 95% CI [−4.2 to −0.02], *p* = 0.046) while for the MS-specific model, this was true for quartile 1 vs quartile 3 (difference = −3.1, 95% CI [−5.6 to −0.59], *p* = 0.008). The improvement of using an MS-specific model was also true for the SDMT (*p* = 0.02, *η*^2^ = 0.013), where quartile groups were again significantly different for both HC-based and MS-specific models. Tukey HSD indicated lower SDMT values for quartile 1 compared with quartile 3 for the HC-based curves (difference = 6.8, 95% CI [2.5–11.1], *p* = 3.4*10^−4^). For the MS-specific quartiles, these were present for quartiles 1 and 3 and quartiles 1 and 4 (difference = 5.9, 95% CI [0.93–10.8], *p* = 0.01, and difference = 6.8, 95% CI [1.7–11.9], *p* = 3.7*10^−3^, respectively), which are demonstrated in Figure 5. Generally, the SDMT values were lower for lower quartiles compared with higher quartiles. No differences between quartiles for both MS-specific and HC-based models were found for the T25FWT.

### Predictive Value of Reference Quartiles

**Figure 6:**
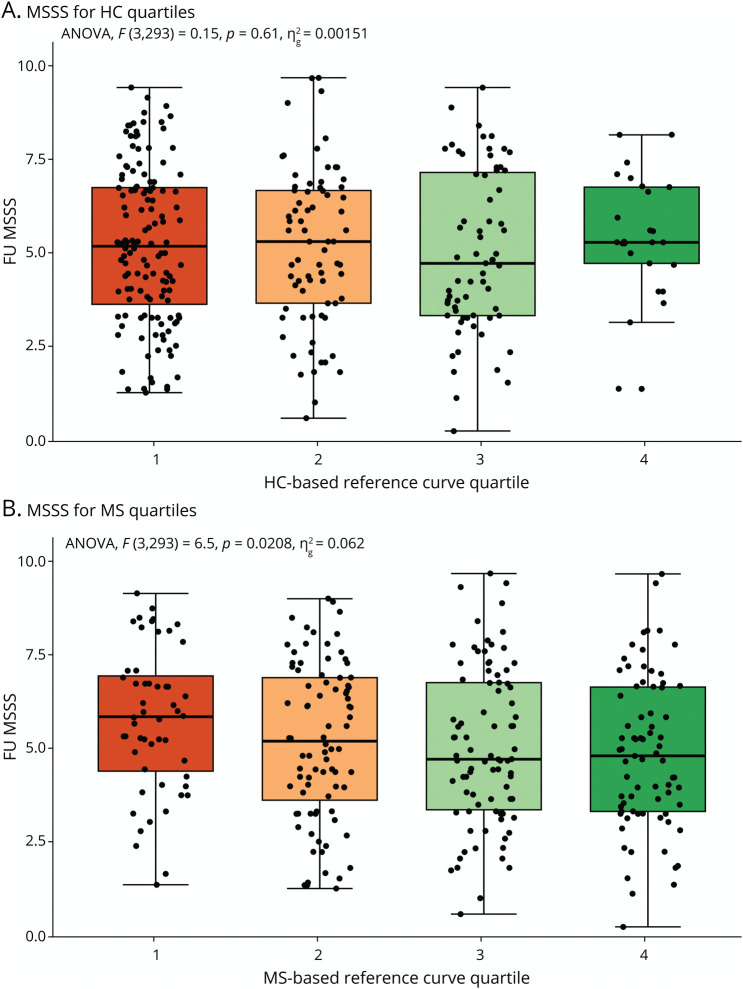
Boxplot of the MSSS at FU for Quartiles Derived From Both HC-Based and MS-Specific Models MSSS scores plotted for the baseline quartiles derived from the HC-based reference curves (A) and the MS-specific reference curves (B). FU = follow-up; HC = healthy control; MS = multiple sclerosis; MSSS = multiple sclerosis severity score.

For the subset with a 5-year longitudinal follow-up, there was a significant improvement of the addition of MS-specific reference curve quartiles at baseline to predict follow-up MSSS compared with HC reference quartiles (*p* = 4.5*10^−4^, *η*^2^ = 0.057). Differences in MSSS at follow-up were found for MS-specific reference curve quartiles (*p* = 0.021, *η*^2^ = 0.062) at baseline. This effect was not present for the HC-based quartiles (*p* = 0.610, *η*^2^ = 0.002), as shown in Figure 6. The estimates again show a decline in FU MSSS with MS-based quartile predictions; however, between-group analyses did not survive Tukey HSD–inherent correction for multiple testing. There was no association of EDSS progression, between the quartiles for both the MS-specific and HC-based curves (*p* = 0.24 and *p* = 0.16, respectively, eFigure 10). However, there was a significant association of cognitive decline for MS-specific quartiles (*p* = 0.041, odds ratio (OR) = 0.74, 95% CI [−0.59 to −0.01]) while this was not present for HC-based quartiles (*p* = 0.11, OR = 0.88, 95% CI [−0.56 to 0.05]). This suggests that for every increase in MS-specific quartile, that is, more brain volume compared with peers, the odds of cognitive decline decrease by approximately 26.4% (eFigure 11).

## Discussion

This study investigated whether MS-specific brain volume models offer improved relevance for explaining disease severity in MS compared with commonly used generic HC-based models. We showed that brain volume quartiles obtained with MS-specific reference offer improved associations with clinical variables such as the MSSS, SDMT score, and 9HPT score. Moreover, we found significant differences between the MS-specific quartiles regarding all clinical outcome measures, except the T25FWT, while clinical associations with HC-based quartiles were only found for the SDMT and 9HPT. These findings were based on a linear model, which was shown to be sufficient to model MS-specific reference curves with the current data set of MRI scans from approximately 1,000 participants. Our findings underscore the importance of incorporating disease-specific factors into quantitative MRI analyses to better understand disease progression and optimize clinical management strategies in MS.

Patients with lower MS-specific quartile scores (i.e., more atrophy than expected) had significantly higher MSSS values, indicating that patients with brain volumes more negatively deviating from the population have worse disease severity. Multiple studies have shown that (loss of) brain volume is related to both cognitive and physical impairment.^28,29^ Our findings underline the importance of MS-specific models because these relations were not present for HC-based models. No differences were observed between progressive MS and RRMS, only after post hoc testing. These differences are likely attributable to the larger sample size of the RRMS group compared with the progressive MS group in the cohort. Moreover, MSSS values at follow-up significantly differed between the MS-specific quartiles at baseline while HC-based quartiles did not differ in MSSS values. Similar cross-sectional and longitudinal relationships between brain volumes and MSSS have also been previously described^30^ although these were based on raw volumes and not models for deviation from a reference population. For cross-sectional analyses with the 9HPT and the SDMT, in both HC-specific and MS-specific models, the lower quartiles were associated with worse outcome on both clinical tests while MS-specific curves improved these associations. For the T25FWT, no associations were found in either model, comparable with a recent study that also did not find relationships between the T25FWT score and atrophy.^31^ There was no association of EDSS progression with the quartiles from MS-specific and HC-based curves. However, this was observed for cognitive decline for the MS-specific curves, which were not present for the HC-based curves.

The most appropriate MS-specific model turned out to be a relatively simple linear regression while more advanced models, using nonlinear and nonparametric fitting, did not provide substantial improvements. This is in line with a recent study that modeled brain volume for the whole human lifespan for healthy individuals using more than 100,000 scans, which showed that a combination of gray and white matter volume generally linearly declines for the age span that was included in our study.^32^ In addition, they showed that the ventricular volume only starts to exponentially increase (which negatively affects brain volume) from older ages,^32^ which are the extremes for the presented data. Similar observations were found for other studies where whole-brain volume and, in particular, gray matter volume, decline linearly from the age of approximately 30 years.^33,34^ Notably, brain charts and a meta-analysis indicated that male patients have larger brain volumes compared with female patients^35^ while the opposite effect was observed in this study. However, several studies have shown that in MS, male patients generally have increased atrophy compared with female patients,^36,37^ which might explain these effects. It must be noted that the estimation of disease duration in this study is based on the reported onset of clinical symptoms, which is commonly used in MS research but may not capture the full biological time line of the disease because MS is believed to begin years before clinical symptoms manifest.^38^ Although disease duration provides essential context for interpreting brain atrophy and its relationship with disease progression, it represents only part of the disease's time line. Novel approaches such as brain age could provide additional information.

Our study showed the value of MS-specific curves for whole-brain volumes, which is a general correlate of progression in MS. However, several studies have shown that regional volumes are more specific for different types of symptoms and may improve association with clinical variables.^29,39^ For example, recent work in large cohorts has shown the thalamus to be the most predictive of EDSS progression^40^ while cortical volumes are most predictive of cognitive decline.^14^ In addition, while the 9HPT has shown to associate strongly with normalized thalamic volume,^41^ the T25FWT most strongly associates with spinal cord damage.^42^ Future studies should investigate the development of region-specific models to further refine the clinical utility of quantitative MRI.

An additional challenge is the incorporation of DMT use into MS-specific models. Previous studies have shown that DMT use influences brain volume measurements,^26,27^ but accounting for these effects is statistically complex because of the heterogeneous treatment pathways in MS. Individuals may start on one DMT and later transition to another, leading to small and underpowered subgroups. We explored DMT use and efficacy across quartiles and found no significant differences, suggesting that the observed relationships between quartiles and clinical outcomes were independent of treatment effects within this cohort. However, this analysis does not capture the full complexity of DMT transitions or variations in timing and efficacy, which may still influence brain volume metrics. Larger data sets could address DMT effects, transitions, and timing of treatment initiation into MS-specific models. Moreover, pseudoatrophy effects should be investigated in future studies, which was not possible to perform here, given the limited participants initiating or switching treatment before the scan.

The findings of this proof-of-concept study potentially have clinical implications for the management of MS because they indicate that our MS-specific brain volume model provides more robust associations with clinical outcomes compared with the HC-based model. This research highlights the necessity of tailoring diagnostic and prognostic tools to the unique characteristics of MS and that MS-specific curves could significantly enhance the precision of disease severity assessment and more precise detection of disease progression. In fact, recent work has indicated that quantitative MRI-based reports have potential to affect daily clinical practice, and based on our study, such reports would significantly benefit from incorporating reference data from MS groups rather than healthy participants.^9^ This would allow clinicians to tailor treatment strategies more effectively, for example, by changing to a DMT that reduces the chance of additional loss of brain volume, rather than waiting for a relapse.^8^ In addition, the impact of therapeutic interventions could be assessed more accurately in trials, as disease factors are taken into account.

While the findings are based on group-level analyses, we recognize the growing need for individualized approaches. The use of quartiles, rather than finer stratifications such as percentiles, was chosen to ensure sufficient sample sizes for robust statistical comparisons, given the data set size; however, future studies with larger data sets could explore finer subdivisions to enhance granularity and potential clinical utility.^24^ An important consideration for the application of MS-specific reference curves is the current variability introduced by differences in imaging software and scanner hardware; hence, cross-scanner reliability remains to be studied. Harmonizing MR images will be critical for translating these findings into tools suitable for individual patient management.^11^ Incorporating additional biomarkers, such as lesion burden, spinal cord involvement, and blood or CSF markers, could complement atrophy measurements.^43-45^ Moreover, integrating imaging metrics and molecular data into models represents the next step toward bridging the gap between group-level research and individualized care. To establish their long-term predictive value, these studies should incorporate longitudinal follow-up and clearly defined clinical end points. This would enable a comprehensive assessment of the sensitivity and specificity of MS-specific quartiles in their applicability in monitoring and predicting clinical progression.

Several limitations should be acknowledged. First, we focused on whole-brain volume only, as discussed above, because this is the most frequently used outcome measure. Second, we did not include lesion metrics or spinal cord involvement in this study because this study specifically focused on atrophy to compare brain volume curves between MS-specific and HC-based models. Third, for this study, only cross-sectional imaging data were used, while a previous study implied that longitudinal models may improve modeling of brain volumes changes.^46^ Fourth, a recent study has shown that people with larger (premorbid) brains may have more brain reserve^47^ while this and other forms of reserve were not accounted for because of nonstandard methodology for quantifying head size. Fifth, the MS-specific curves derived from our cohort were compared with HC curves generated from external public data sets. This methodology could introduce bias because the MS-specific curves may be tailored to the unique characteristics of our study population, potentially decreasing variability and leading to improved performance within the cohort. Finally, the reliance on a single-center cohort may limit the generalizability of our results. Incorporating larger external MS cohorts, such as clinical trial data sets, with diverse imaging protocols and scanner types would enhance the homogeneity and reliability of the results and account for effects of treatment.

In conclusion, our study underscores the importance of MS-specific curves to explain disease severity in MS, which could become a valuable tool in MS management. By accounting for disease-specific factors, these curves provide a more accurate representation of disease severity compared with HC-based curves. These models were relevant for multiple clinical outcome measures and longitudinal progression. Future work is now required to evaluate the feasibility of implementing such models in clinical routine, to improve clinical management of people with MS.

## Glossary

9HPT: 9-Hole Peg Test
AIC: Akaike information criterion
DMT: disease-modifying therapy
EDSS: Expanded Disability Status Scale
FA: flip angle
HC: healthy control
ICV: intracranial volume
MS: multiple sclerosis
MSSS: multiple sclerosis severity score
NBV: normalized brain volume
PPMS: primary progressive MS
pwMS: people with multiple sclerosis
RCI: reliable change index
RRMS: relapsing remitting MS
SDMT: Symbol Digit Modalities Test
SPMS: secondary progressive MS
T25FWT: Timed 25-Foot Walk Test
TE: echo time
TI: inversion time
TR: repetition time
